# Feasibility and safety of totally endoscopic Bentall procedure via right anterior mini-thoracotomy: Early experience

**DOI:** 10.1016/j.xjtc.2026.102277

**Published:** 2026-02-06

**Authors:** Marwan Hamiko, Saad Salamate, Ali Bayram, Julia Rogaczewski, Jacqueline Kruse, Miriam Silaschi, Kaveh Eghbalzadeh, Ali El-Sayed Ahmad, Farhad Bakhtiary

**Affiliations:** Department of Cardiac Surgery, University Heart Center Bonn, Bonn, Germany

**Keywords:** totally endoscopic surgery, RAMT, aortic root surgery

## Abstract

**Objectives:**

Minimally invasive aortic root surgery via right anterolateral minithoracotomy (RAMT) is a developing technique that has shown promise in selected patient populations. This study reports our initial experience with the totally endoscopic Bentall procedure performed through RAMT using 3-dimensional visualization.

**Methods:**

We retrospectively analyzed clinical data from 35 patients who underwent a totally endoscopic Bentall procedure via RAMT between March 2023 and July 2025. Collected variables included operative and aortic crossclamp time, cardiopulmonary bypass duration, ventilation time, postoperative drainage, intensive care unit and hospital length of stay, as well as midterm follow-up outcomes.

**Results:**

The mean patient age was 61.3 ± 12.0 years, with 74.3% being male. Mean procedure time was 153.0 minutes (interquartile range [IQR], 140.0-183.0), with mean cardiopulmonary bypass and aortic crossclamp times of 116.6 ± 26.7 minutes and 85.0 ± 22.5 minutes, respectively. No conversions to sternotomy or re-thoracotomies were required. Mean chest drainage in the first 24 hours was 629.3 ± 240.8 mL. Mean ventilation time was 7.0 ± 5.9 hours, median intensive care unit stay was 24.0 hours (IQR, 21.0-38.0), and hospital stay was 6.0 days (IQR, 5.0-8.0). Thirty-day mortality was zero. All patients survived during the median follow-up period of 18 months, and cosmetic results were consistently excellent.

**Conclusions:**

The totally endoscopic Bentall procedure via RAMT is a feasible and safe minimally invasive technique in selected patients. When performed by experienced surgical teams in high-volume centers, this approach offers favorable operative and midterm outcomes without compromising safety or effectiveness.

## Graphical Abstract

**Figure:**
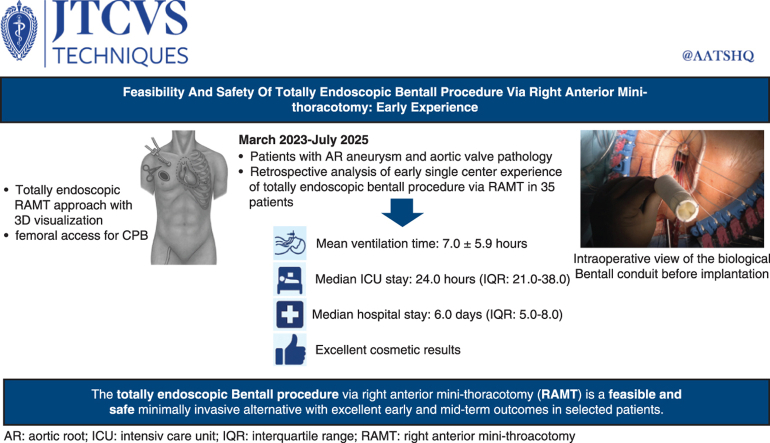

**Figure:**
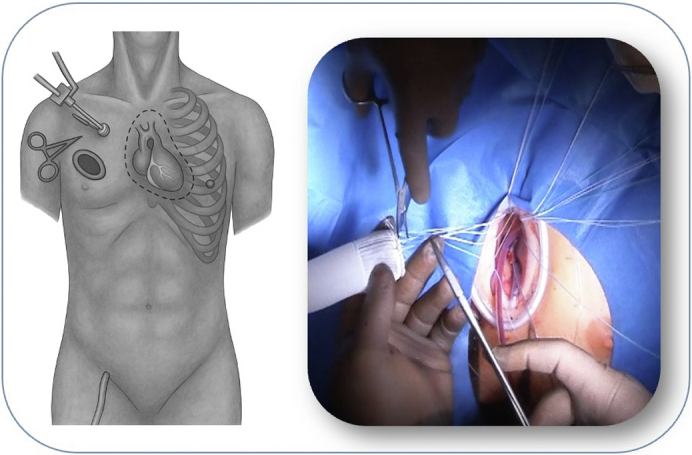
Totally endoscopic 3D-guided Bentall procedure via right anterior minithoracotomy.

**Central Message**

The totally endoscopic Bentall procedure via right anterior minithoracotomy is a feasible and safe minimally invasive alternative with excellent early and midterm outcomes in selected patients.

**Perspective**

As surgical expertise and endoscopic technologies continue to evolve, the totally endoscopic Bentall procedure via right anterior minithoracotomy may become a valuable minimally invasive alternative to conventional approaches in selected patients, potentially expanding the boundaries of complex aortic root surgery.

With ongoing advancements in surgical techniques and perioperative technologies, minimally invasive cardiac surgery has become increasingly adopted across a broadening spectrum of cardiac procedures.^1^^,^^2^ Traditionally, full median sternotomy has been the gold standard for aortic root (AR) replacement, particularly in the Bentall procedure.^3^^,^^4^ However, the surgical landscape is shifting toward less-invasive approaches, driven by patient demand, improved instrumentation, and growing surgeon expertise. Among these approaches, the totally endoscopic Bentall procedure via right anterior minithoracotomy (RAMT) has emerged as a promising alternative.^5^, ^6^, ^7^, ^8^, ^9^ This technique, enabled by video assistance and the use of advanced technologies such as automated suturing systems, has demonstrated feasibility and safety in selected patient cohorts, offering favorable outcomes with shorter length of stay in the intensive care unit (ICU) and in the hospital, decreased postoperative pain, faster pulmonary recovery, improved cosmetic results, and earlier return to daily activities.^7^^,^^10^ These benefits raise the question of whether a similar approach could be safely and effectively applied to more complex procedures such Bentall procedure.

At our institution, totally endoscopic approach through RAMT for aortic valve replacement became a standard approach since 2017. Building upon this experience, we extended the use of this technique to include the Bentall procedure in select patients. The aim of this study is to present our initial clinical experience with this technique and to evaluate its feasibility and safety.

## Methods

### Study Population

**Figure 1:**
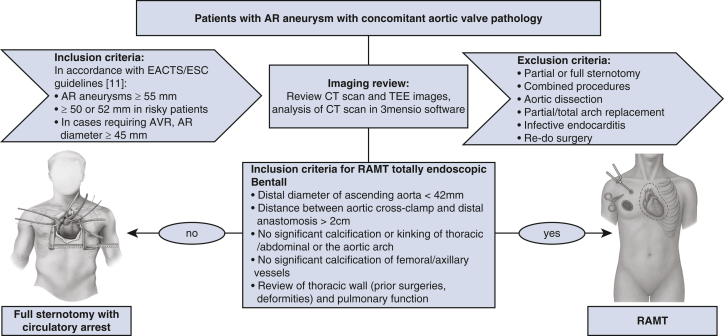
Patient selection and screening algorithm for totally endoscopic Bentall procedure via RAMT. *RAMT*, Right anterior minithroacotomy; *AR*, aortic root; *EACTS*, European Association for Cardio Thoracic-Surgery; *STS*, Society of Thoracic Surgeons; *AVR*, aortic valve replacement; *CT*, computed tomography; *TEE*, transesophageal echocardiography.

This retrospective study included all adult patients (≥18 years) who underwent a totally endoscopic Bentall procedure via RAMT at our institution between March 2023 and July 2025. Ethical approval was obtained from the University of Bonn (approval number 464/22; December 28, 2022), and all patients provided written informed consent. Preoperative patient selection, as well as inclusion and exclusion criteria for the totally endoscopic RAMT Bentall procedure, were performed according to a standardized institutional screening algorithm and are presented as a flowchart (Figure 1). Indication for surgery followed current European Society of Cardiology/European Association for Cardio-Thoracic Surgery guidelines.^11^ Preoperative computed tomography (CT) scan and analysis was mandatory and conducted for all patients using the 3mensio software (Pie Medical Imaging) for surgical planning, including anatomical assessment, especially the spatial relationship between the aorta and the chest wall, and access for the cardiopulmonary bypass (CPB) via femoral or axillary vessels.

Baseline characteristics, operative data, and perioperative outcomes were collected from a standardized institutional database. Preoperative risk was assessed using the European System for Cardiac Operative Risk Evaluation II, and clinical status was categorized using the New York Heart Association classification.

### Surgical Technique

**Figure 2:**
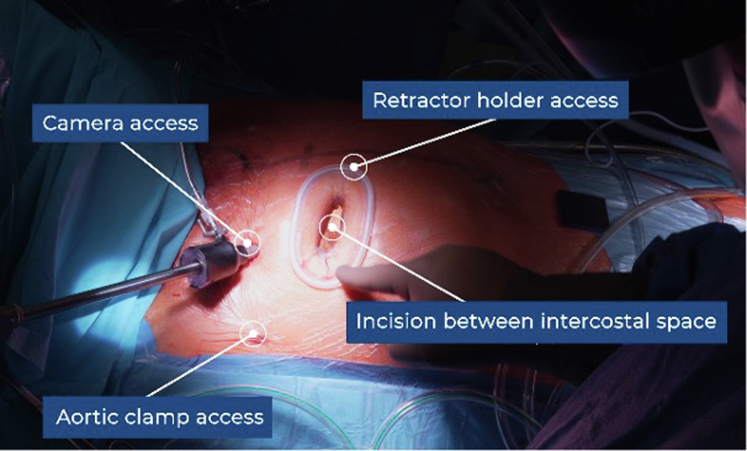
Surgical approach showing the skin incision in third intercostal space (*ICS*) with insertion of the soft-tissue retractor. Two additional access for 3-dimensional camera port and aortic clamp in the second ICS.

**Figure 3:**
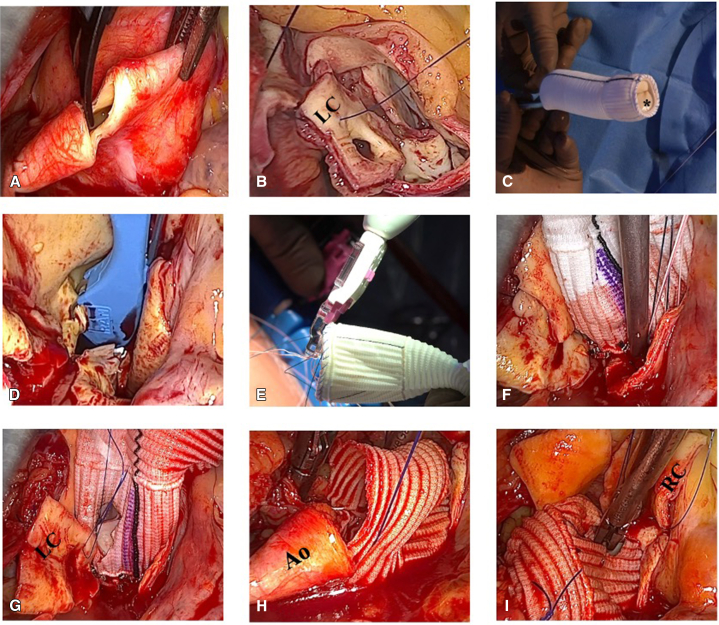
Step-by-step surgical procedure A, Resection of the aneurysm, leaving a 2-cm cuff for distal anastomosis. B, Coronary buttons were cut and retracted using stay sutures. C, Biological conduit consisting of vascular graft and aortic valve prosthesis (∗). D, Annular sutures were placed using the RAM suture device. E, Sew-Easy cassettes placed on the Sew-Easy device, passing the sutures through the sewing cuff of the conduit, completing the horizontal mattress stitches. F, The Cor-Knot Mini device was used to secure the ring sutures by crimping a titanium fastener and automatically trimming the suture tails. G, Reimplantation of the LC. H, Performing distal anastomosis between remaining ascending aorta (*Ao*) and the vascular prosthesis using a running 4-0 polypropylene suture. I, Reimplantation of the right coronary artery (*RC*). *LC*, Left coronary artery.

The patient is positioned supine, with defibrillator pads in place. After induction of general anesthesia and intubation, transesophageal echocardiography (TEE) is used for intraoperative monitoring. Percutaneous femoral cannulation is performed under ultrasound guidance using the Seldinger technique. A dual-stage venous cannula (23-25 Fr) is advanced to the superior vena cava, and an 18-Fr arterial cannula is inserted into the right femoral artery. Guidewire and cannula positioning are confirmed via TEE. After the initiation of CPB, a 3- to 4-cm RAMT is performed in the third intercostal space, without rib resection or mammary vessel transection. A soft-tissue retractor (ValveGate Soft Tissue Protector) is used instead of a rib spreader. The procedure is performed entirely endoscopically under 3-dimensional (3D) visualization. Two additional 5-mm ports are placed in the second third intercostal space for the 3D camera (Aesculap EinsteinVision) and the Chitwood aortic clamp (Scanlan International, Inc) (Figure 2). The pericardium is opened, the phrenic nerve is preserved, and stay sutures are placed. A left ventricular vent catheter is inserted via the right superior pulmonary vein. The ascending aorta and pulmonary artery are dissected for placement of the Chitwood clamp. A cardioplegia catheter is introduced into the ascending aorta. Myocardial protection is achieved with Custodiol crystalloid solution (Koehler Chemie). After crossclamping, the aneurysmal ascending aorta is resected, leaving a 2-cm cuff for distal anastomosis (Figure 3, *A*). The coronary buttons are mobilized and retracted with stay sutures (Figure 3, *B*), and the aortic sinuses are excised. The native valve leaflets are removed, and the annulus is sized. A biological conduit (Vascutek Valsalva graft prosthesis; Vascutek Terumo Inc) is prepared with a sewn-in bioprosthetic valve using a running 3-0 polypropylene suture (Figure 3, *C*). The conduit was sewn during the aortic crossclamp time. Annular sutures are placed either manually with pledgeted 2-0 polyester sutures or using the automated RAM COR-SUTURE QUICK LOAD device (LSI Solutions) (Figure 3, *D*). The device places double-bite mattress sutures with dual curved needles. Sutures are passed through the conduit's sewing cuff using the Sew-Easy system (LSI Solutions) (Figure 3, *E*), then grouped, tensioned, and secured using the Cor-Knot Mini device (LSI Solutions) (Figure 3, *F*). The use of these systems is not mandatory for performing the procedure but is an optional tool system to simplify suture placement, reduce technical challenges, and minimize ischemic times. The coronary buttons are reimplanted using 5-0 polypropylene sutures, beginning with the left coronary artery (Figure 3, *G*). The distal anastomosis between the graft and native aorta is performed with 4-0 polypropylene suture (Figure 3, *H*), followed by reimplantation of the right coronary artery (Figure 3, *I*). Before aortic unclamping, temporary epicardial pacing wires are placed. The graft and left ventricle are deaired under TEE guidance. Prosthesis function, ventricular performance, and coronary flow are confirmed. After CPB weaning, decannulation is performed, and the femoral artery is closed using conventional vascular closure system. Pleural and pericardial drains are placed. The thoracotomy is closed in layers, the ribs are secured with 2 FiberWire sutures (Arthrex), and the skin incision closed with absorbable subcutaneous sutures. An educational video of the procedural steps is provided as supplementary material (Video 1).

### Postoperative Management

After surgery, all patients were transferred to the ICU for postoperative monitoring and care. Early extubation and transfer to intermediate care were pursued when clinically feasible; some patients were extubated directly in the operating room as part of a fast-track protocol. Chest tube output was closely monitored at set intervals within 24 hours. In accordance with institutional guidelines, significant bleeding was defined as >400 mL/h in the first 3 hours or need for rethoracotomy due to hemodynamic instability. Operative metrics and clinical outcomes, including ventilation time, ICU/hospital stay, rethoracotomy, and both in-hospital and 30-day mortality, were recorded.

### Follow-up

Structured telephone interviews were conducted at 1, 3, and 6 months and 1, 3, and 5 years postoperatively. Follow-up focused on patient survival and the occurrence of any aortic reinterventions.

### Statistical Analysis

All statistical analyses were descriptive and conducted using SPSS, version 29.0 (SPSS Inc) and GraphPad Prism, version 8.4.3. No hypothesis testing was performed because of the study's observational nature. Categorical variables are reported as frequencies and percentages; continuous variables as mean ± standard deviation or median with interquartile range (IQR), on the basis of distribution assessed via QQ plots. Kaplan-Meier analysis was used to estimate cumulative survival, with curves illustrating 30-day and 1-year mortality.

## Results

### Study Population

Baseline characteristics, including preoperative echocardiographic and CT morphologic measurements, comorbidities, and presenting symptoms of the 35 included patients, are shown in Table 1. All underwent a totally endoscopic Bentall procedure using a RAMT approach with 3D visualization. Of these, 26 (74.3%) were male, with a mean age of 61.3 ± 12.0 years. The median European System for Cardiac Operative Risk Evaluation II was 2.4 (IQR, 1.9-2.7), indicating moderate surgical risk. Preoperative echocardiography showed combined aortic valve disease in 23 patients (65.7%), isolated stenosis in 6 (17.1%), and isolated regurgitation in 6 (17.1%). AR diameter ranged from 45 to 55 mm (median, 52.0 mm). At the time of surgery, 51.4% of patients presented with New York Heart Association class II symptoms.

**Table 1:** Baseline characteristics

Variable	Value
Patient characteristics	
Number of patients	35
Age, y, mean ± SD	61.3 ± 12.0
Male sex	26 (74.3%)
BMI, kg/m^2^, mean ± SD	27.3 ± 4.7
EuroSCORE II, %, median, IQR	2.4 [1.9, 2.7]
Aortic valve pathology	
Stenosis	6 (17.1%)
Regurgitation	6 (17.1%)
Combined	23 (65.7%)
Bicuspid valve	16 (45.7%)
Left ventricular ejection fraction	
>50%	34 (97.1%)
30%-50%	1 (2.9%)
<30%	0 (0.0%)
Mean aortic root diameter, mm, median [IQR]	52.0 [49.0, 54.0]
Concomitant disease and cardiovascular risk factors	
Hypertension	31 (88.6%)
Hyperlipidemia	9 (25.7%)
Diabetes	4 (11.4%)
Smoker	2 (5.7%)
Coronary artery disease	4 (11.4%)
COPD	1 (2.9%)
Atrial fibrillation	7 (20.0%)
PVD	0 (0.0%)
CVD	1 (2.9%)
Previous stroke	2 (5.7%)
Chronic renal failure	0 (0.0%)
NYHA	
I	3 (8.6%)
II	18 (51.4%)
III	14 (40.0%)

*SD*, Standard deviation; *BMI*, body mass index; *EuroSCORE II*, European System for Cardiac Operative Risk Evaluation II; *IQR*, interquartile range; *COPD*, chronic obstructive pulmonary disease; *PVD*, peripheral vascular disease; *CVD*, cerebrovascular disease; *NYHA*, New York Heart Association.

### Intraoperative Outcome

Detailed intraoperative outcomes are summarized in Table 2. In all patients included in the present series, the aneurysm was completely resected, and no aneurysmal remnants were left. In 17 cases, annular sutures were placed using the RAM device (RAM COR-SUTURE QUICK LOAD), whereas in the remaining 18 cases the sutures were placed manually. The Cor-Knot Mini device was used in all cases. The median procedure duration was 153.0 minutes (IQR, 140.0-183.0 minutes). Mean CPB and aortic crossclamp times were 116.6 ± 26.7 and 85.0 ± 22.5 minutes, respectively. No patient required circulatory arrest for distal anastomosis. In all 6 patients with isolated aortic insufficiency, the native valve was unsuitable for valve-sparing root replacement (David procedure). Findings included multiple fenestrations (n = 3), thickened and retracted cusps (n = 2), and partial sclerosis (n = 1), making durable valve reconstruction unfeasible. All patients received a biological AR conduit; no mechanical conduits were used. Patients younger than 60 years had explicitly requested biological valves, and these preferences were respected during surgical planning.

**Table 2:** Intraoperative characteristics

Variable	Value
Procedure time, min, median [IQR]	153.0 [140.0, 183.0]
CPB time, min, mean ± SD	116.6 ± 26.7
Aortic clamp time, min, mean ± SD	85.0 ± 22.5
Type of conduit	
Biological	35 (100.0%)
Mechanical	0 (0.0%)

*IQR*, Interquartile range; *CPB*, cardiopulmonary bypass; *SD*, standard deviation.

### Postoperative Outcome

Postoperative outcomes are shown in Table 3. No major postoperative complications occurred. There were no cases of significant bleeding, conversion to sternotomy, myocardial infarction, stroke, or acute renal failure. Delirium occurred in 1 patient (2.9%). Atrial fibrillation was the most common arrhythmia (4 cases, 11.4%). One patient (2.9%) required a permanent pacemaker due to third-degree AV block. Pneumonia developed in 4 patients (11.4%), without need for reintubation. No wound infections occurred, and cosmetic results were excellent. Both 30-day and in-hospital mortality were zero.

**Table 3:** Postoperative outcome

Variable	Value
Complications	
Re-thoracotomy	0 (0.0%)
Conversion to full sternotomy	0 (0.0%)
Disabling stroke	0 (0.0%)
Postoperative delirium	1 (2.9%)
Acute kidney injury	0 (0.0%)
Myocardial infarction	0 (0.0%)
Postoperative atrial fibrillation	4 (11.4%)
Pacemaker implantation	1 (2.9%)
Pneumonia	4 (11.4%)
Reintubation	0 (0.0%)
Wound infection	0 (0.0%)
Complication to CPB access	0 (0.0%)
Mortality	
30-d mortality	0 (0.0%)
In-hospital mortality	0 (0.0%)
Blood loss 24 h postoperative, mL, mean ± SD	629.3 ± 240.8
Ventilation time, min, mean ± SD	7.0 ± 5.9
ICU time, h, median [IQR]	24.0 [21.0, 38.0]
Time to discharge, d, median [IQR]	6.0 [5.0, 8.0]

*CPB*, Cardiopulmonary bypass; *IQR*, interquartile range; *SD*, standard deviation; *ICU*, intensive care unit.

Mean 24-hour drainage volume was 629.3 ± 240.8 mL. Mean ventilation time was 7.0 ± 5.9 hours. ICU stay had a median duration of 24 hours (IQR, 21.0-38.0 hours), and hospital discharge occurred after a median of 6 days (IQR, 5.0-8.0 days).

### Follow-up

All 35 patients completed follow-up (median 18 months; range, 1-28 months). No structural valve deterioration, reintervention, or mortality occurred.

## Discussion

Minimally invasive cardiac surgery has gained widespread acceptance worldwide and continues to show an expanding trend in contemporary practice. An increasing number of cardiac surgery centers are now using the RAMT approach to perform complex cardiac procedures.^9^^,^^12^, ^13^, ^14^, ^15^, ^16^, ^17^ The fundamental objective of minimally invasive techniques is to avoid full sternotomy and its associated complications.^12^^,^^18^, ^19^, ^20^ Although CPB and thoracotomy are still required, the procedure is considered minimally invasive in terms of surgical access. Through a 3- to 4-cm RAMT incision, the thoracic cage remains intact without rib resection or the use of a rib spreader, thereby minimizing musculoskeletal trauma. The operation is performed entirely endoscopically and aligns with the principles of minimally invasive surgery. Recently, RAMT has become an increasingly established approach for minimally invasive aortic valve surgery.^12^, ^13^, ^14^, ^15^, ^16^ Studies shown its advantages over complete or partial sternotomy. By avoiding sternotomy, patients experience less postoperative pain, faster recovery and mobilization, shorter ICU and hospital stays, and earlier return to normal daily and professional activities. In addition, the cosmetic outcome is markedly improved. These benefits translate into greater patient satisfaction, reduced morbidity, and more efficient resource use within the health care system.^12^^,^^16^^,^^18^^,^^20^, ^21^, ^22^

As surgical expertise and technology have evolved, interest has grown in applying RAMT to more complex procedures such as AR replacement. However, the approach presents challenges: a limited working space, restricted visualization, and a steep learning curve may prolong operative and ischemic times.^7^^,^^23^ These can be mitigated through high-definition 3D visualization, advanced instruments, and standardized techniques that maintain stable exposure while minimizing incision size.^9^^,^^21^^,^^24^ The use of high-definition, 3D endoscopic camera systems provide clear and detailed visualization, as well as precise suturing, and conduit implantation even through a small access incision, when performed by experienced surgeons.

Despite these advantages, reports on RAMT for AR surgery remain limited. Owing to technical complexity, many surgeons continue to favor open approaches. Traditionally, minimally invasive access to the AR has involved upper partial sternotomies using T-, J-, or other modified incisions.^25^, ^26^, ^27^, ^28^, ^29^ These alternative approaches have demonstrated safety and feasibility, with reduced bleeding, shorter ventilation, and faster recovery compared with full sternotomy.^12^^,^^18^, ^19^, ^20^

The present series of selected 35 patients represents, to our knowledge, one of the larger analyses of the totally endoscopic Bentall procedure via RAMT with 3D visualization. Conventional surgical instruments and advanced suturing techniques were used, and distal anastomosis was performed without circulatory arrest. No rib resections were necessary, and the right internal mammary artery was preserved in all cases. Although this study was not designed as a matched comparison with sternotomy, it demonstrates that totally endoscopic AR surgery can be performed safely in selected patients by surgeons experienced in minimally invasive valve procedures. Our results add to the growing body of literature supporting the technical feasibility and noninferiority as well as safety of this approach.

Previous studies by Johnson and colleagues^5^^,^^8^ and Ji and colleagues^6^ have shown that minimally invasive Bentall procedures via RAMT are feasible and yield satisfactory outcomes. Johnson and colleagues^5^^,^^8^ reported 7 cases using video-assisted visualization and partial circulatory arrest, with mean CPB and aortic crossclamp times of 202.9 and 161.9 minutes, respectively. Ji and colleagues^6^ described 15 cases using direct visualization through a 6-cm incision with rib spreading, reporting CPB and crossclamp times of 138.5 and 95.0 minutes. In our study, mean CPB and crossclamp times were 116.6 and 85.0 minutes, respectively. These results likely reflect increased institutional experience, procedural standardization, and the use of automated suturing and fastening devices such as the RAM and Cor-Knot systems. The use of these systems is optional but can simplify suture placement, reduce technical challenges and ischemic times, and improve ergonomics and reproducibility without affecting valve function. In this series, the device was used to enhance efficiency while maintaining surgical precision and suturing quality. Historically, prolonged CPB and crossclamp times have been considered limitations of the RAMT approach compared with conventional sternotomy.^30^ Regarding operative times, in our series we observed a trend to operative time reduction over the study period, reflecting growing team experience, process standardization and optimization, and the implementation of automated suturing and fastening systems.

In our series, postoperative outcomes were favorable and comparable with, or slightly better than, previously published data. Mean ventilation time was 7.0 hours, shorter than the 10.6 and 12.5 hours reported by Johnson and colleagues^5^ and Ji and colleagues,^6^ respectively. Median ICU stay was 24.0 hours, closely comparable with Johnson and colleagues’ 31.8 hours and Ji and colleagues’ 36.0 hours. Median hospital discharge occurred after 6.0 days, in line with Ji and colleagues’ 5.8 days.^6^ Importantly, no rethoracotomies, cerebrovascular events, or 30-day mortalities were observed. During a median follow-up of 18.2 months, no reinterventions were necessary, supporting the safety and durability of the procedure in appropriately selected patients.

Our study supports growing evidence that the totally endoscopic RAMT approach is a safe and effective technique for selected patients undergoing AR surgery. Given its novelty and technical complexity, careful patient selection remains essential. Totally endoscopic AR procedures should currently be performed only in highly specialized centers with extensive experience in both conventional and totally endoscopic cardiac surgery. At our institution, surgeons follow a stepwise training pathway: initial experience with conventional Bentall procedures is followed by totally endoscopic valve surgery via RAMT, before progressing to totally endoscopic AR surgery. This structured progression ensures safety and reproducibility. Implementation of the RAMT technique with 3D visualization demonstrates its potential to achieve outcomes comparable with conventional sternotomy approaches. In specialized centers, RAMT may optimize resource use without compromising quality or patient safety. Ultimately, the adoption of new surgical techniques should prioritize perioperative safety and favorable mid- to long-term outcomes.

### Limitations

This study has several limitations. Its retrospective, nonrandomized design entails a risk of selection bias and limits causal inference. All procedures were performed by a single, highly experienced surgeon, which may affect generalizability. The technique demands advanced proficiency in minimally invasive and conventional Bentall procedures, confining its current application to specialized centers with extensive endoscopic cardiac surgery expertise. Broader implementation will require structured training, technological refinement, and validation through multicenter collaboration. Future prospective, multicenter, randomized investigations—particularly those comparing conventional approaches—are necessary to substantiate these findings and assess their generalizability across varying institutional and operator experience levels. Follow-up in this study was based on telephone interviews, which may not fully capture postoperative valvular function or detailed aortic-related outcomes due to the lack of systematic echocardiographic or CT imaging.

## Conclusions

The current findings indicate that RAMT with 3D visualization and endoscopic assistance represents a safe and viable surgical approach for AR replacement. When performed by an experienced team in a center of excellence, this minimally invasive technique offers favorable clinical outcomes without compromising surgical precision or patient safety. The totally endoscopic Bentall procedure via RAMT appears to be a suitable alternative for selected patients, with low rates of perioperative morbidity and mortality.

### Webcast

You can watch a Webcast of this AATS meeting presentation by going to: https://www.aats.org/resources/totally-endoscopic-bentall-pro-9863.

**Figure:**
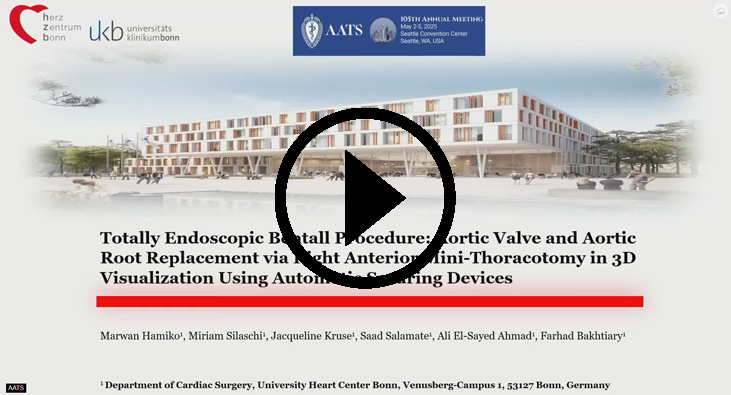

### Audio

You can listen to the discussion audio of this article by going to the supplementary material section below.

## Conflict of Interest Statement

Dr Bakhtiary reports relationships with Edwards Lifesciences, Medtronic, Corcym, and Abbott that include consulting or advisory and speaking fees and a relationship with LSI that includes speaking fees. All other authors reported no conflicts of interest.

The *Journal* policy requires editors and reviewers to disclose conflicts of interest and to decline handling or reviewing manuscripts for which they may have a conflict of interest. The editors and reviewers of this article have no conflicts of interest.

## Supplementary Data

### Audio

Audio 1 This is an audio recording of the presentation of the associated abstract and discussion. The abstract presentation ends at 10:06 and the discussion begins at 10:11.

Video 1 Step-by-step demonstration of the endoscopic right anterior minithoracotomy (RAMT) Bentall procedure. The video illustrates patient positioning, port placement, cardiopulmonary bypass setup, aortic crossclamping, valve and graft preparation, and sequential anastomoses. Key intraoperative views highlight the exposure and technical aspects specific to the endoscopic RAMT approach. This video is intended for educational purposes and to facilitate training in minimally invasive aortic root surgery. Narration: Generated using Google Cloud Text-to-Speech (Chirp 3: HD voices) with custom pronunciation settings for technical terms. Video available at: https://www.jtcvs.org/article/S2666-2507(26)00084-2/fulltext.

Video 2 Video available at: https://www.jtcvs.org/article/S2666-2507(26)00084-2/fulltext.
