# Supplementary figures and images for: Feasibility and safety of totally endoscopic Bentall procedure via right anterior mini-thoracotomy: Early experience
Source: JTCVS Tech. 2026 Feb 6;36:102277. doi: 10.1016/j.xjtc.2026.102277 (PMC13069558; doi:10.1016/j.xjtc.2026.102277)

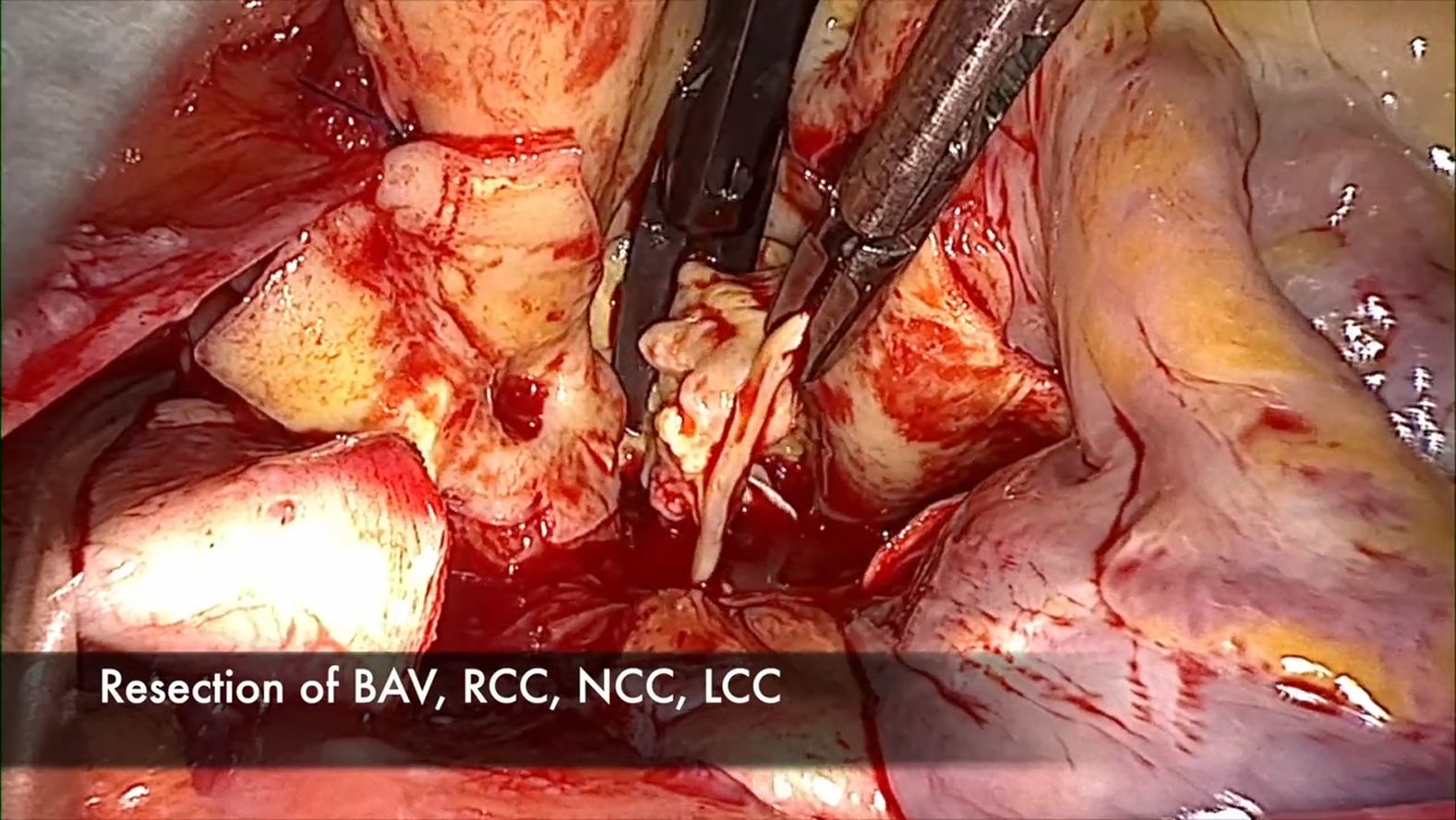

Supplement: Video 1 — Step-by-step demonstration of the endoscopic right anterior minithoracotomy (RAMT) Bentall procedure. The video illustrates patient positioning, port placement, cardiopulmonary bypass setup, aortic crossclamping, valve and graft preparation, and sequential anastomoses. Key intraoperative views highlight the exposure and technical aspects specific to the endoscopic RAMT approach. This video is intended for educational purposes and to facilitate training in minimally invasive aortic root surgery. Narration: Generated using Google Cloud Text-to-Speech (Chirp 3: HD voices) with custom pronunciation settings for technical terms. Video available at: https://www.jtcvs.org/article/S2666-2507(26)00084-2/fulltext. [file fx3.jpg]
